# A Convenient Synthesis of Triflate Anion Ionic Liquids and Their Properties

**DOI:** 10.3390/molecules17055319

**Published:** 2012-05-07

**Authors:** Nikolai V. Ignat’ev, Peter Barthen, Andryi Kucheryna, Helge Willner, Peter Sartori

**Affiliations:** 1 Merck KGaA, PM-ABE, Ionic Liquids Research Laboratory, Frankfurter Str. 250, Darmstadt D-64293, Germany; 2 Inorganic Chemistry, Henrich-Heine-University Düsseldorf, Universitätsstrasse 1, Düsseldorf D-40225, Germany; Email: peter.barthen@uni-duesseldorf.de; 3 Inorganic Chemistry, Bergische University Wuppertal, Gauss Str. 20, Wuppertal D-42119, Germany; Email: andriy.kucheryna@uni-wuppertal.de (A.K.); willner@uni-wuppertal.de (H.W.); 4 Professor Emeritus, Inorganic Chemistry, University Duisburg-Essen, Lotharstrasse 1, Duisburg D-47048, Germany; Email: peter.sartori@online.de

**Keywords:** methyl triflate, ethyl triflate, ionic liquids, viscosity

## Abstract

A solvent- and halogen-free synthesis of high purity triflate ionic liquids via direct alkylation of organic bases (amines, phosphines or heterocyclic compounds) with methyl and ethyl trifluoromethanesulfonate (methyl and ethyl triflate) has been developed. Cheap and non-toxic dimethyl and diethyl carbonate serve as source for the methyl and ethyl groups in the preparation of methyl and ethyl triflate by this invented process. The properties of ionic liquids containing the triflate anion are determined and discussed.

## 1. Introduction

Ionic liquids (ILs) with the triflate anion ([CF_3_SO_3_]^−^; OTf) are of interest for practical application in various fields [1,2,3,4]. Triflate ionic liquids are hydrolytically stable and in many cases they are the preferred reaction media in comparison to ionic liquids with the hydrolytically unstable [PF_6_]^−^ or [BF_4_]^−^ anions. For example, the Claisen rearrangement of aromatic allyl esters in [EtDBU][OTf] results in the formation of 2,3-dihydrobenzofuran derivatives in up to 91% yield. The same reaction in [BMIM][PF_6_] or [BMIM][BF_4_] gives only 9–12% yield [5]. Recently it was demonstrated that [BMIM][OTf] is also a suitable reaction medium for fluorolactonization of unsaturated carboxylic acids [6]. The combination of [BMIM][OTf] with triflic acid (CF_3_SO_3_H, cat.) provides good conditions for the Schmidt reaction, *i.e.*, the conversion of aryl alkyl ketones to the corresponding carboxamides [7]. In triflate ionic liquids polymerization of methyl methacrylate (MMA) generally yields the highest molecular weight [8]. These examples demonstrate the utility of triflate ionic liquids in organic synthesis or in polymer chemistry, but there are further applications for triflate ILs. Due to their high thermal and electrochemical stability these ionic liquids are of interest as heat transfer materials, lubricants and as components of electrolytes for electrochemical cells. The purity of ionic liquids is particularly important for their use in electrochemistry, and quality control should be always in the focus during the preparation of ILs for this application [2].

Typically triflate ionic liquids are prepared by means of the metathesis reaction of the corresponding chlorides, bromides or methylsulfates with metal triflates, CF_3_SO_2_OM, for instance M = K [9,10], Na [11], Li [12], Ag [13], with ammonium triflate [14] or triflic acid, CF_3_SO_3_H [15]. The ionic liquids prepared by means of this methodology have to be purified from the side products: metal chlorides or bromides, or HCl. The side products then need to be disposed of. The reactions referred above were carried out in organic solvents such as dichloromethane, chloroform, acetone, or methanol, but if we are considering ionic liquids as replacements for volatile organic solvents to perform chemical reactions, then logically the ionic liquids themselves should be produced without the use of organic solvents [16]. Mikami wrote “It is also important to underline that to justify the green solvents label, ionic liquids have to be prepared in an ecological way, without the generation of a large amount of waste material” [17].

Recently, the syntheses of ionic liquids under solvent free conditions were tested by application of microwave (MW), power ultrasound (US) and combined MW/US irradiation [18,19]. The authors have carried out the one pot synthesis of triflate ionic liquids by reacting pyridine or *N*-methylpyrrolidine, or *N*-methylimidazole with 1-chlorobutane or 1-chlorooctane in the presence of potassium triflate, KOTf. The reaction mixture was heated under MW-irradiation up to 180 °C after an initial step (10–15 min at 120 °C). This reaction gives the corresponding ionic liquids in 60% to 79% yield [18]. KCl is formed as a side product in this reaction. Replacement of 1-chloroalkanes by 1-bromoalkanes reduced the reaction temperature to 80–90 °C [19]. The yield of ionic liquids increased up to 90%, but KBr is formed as a side product. The final products prepared by means of this methodology have to be purified using organic solvents (acetone, dichloromethane, diethyl ether) to reduce the halogen (chloride or bromide) impurities [18,19]. 

According to an assessment recently carried out by Deetlefs and Seddon [20] applications of microwave (MW), ultrasound (US) or combined MW/US irradiation to the syntheses of ionic liquids do not meet the principles of green chemistry to the full extent. This technology provides only low-medium atom economy and has a poor *E*-factor value. Preparation of ionic liquids by alkylation of organic substances with a suitable alkylating reagent is much “greener” in comparison to the metathesis protocol. 

**Scheme 1:**
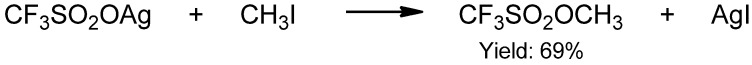
Synthesis of methyl triflate by the reaction of silver triflate with methyl iodide [23].

Trifluoromethanesulfonic acid (triflic acid, CF_3_SO_3_H) is a very strong acid (H_0_ = −14.1 at 22 °C) [21]. It has a broad application as a catalyst [22] in Friedel-Crafts alkylation and acylation, Diels-Alder reactions, aldol condensations, hydrogenation of alkenes, cyclization and polymerization processes. Triflic acid is the starting material for the production of all variety of triflates, *i.e.*, metal salts, organic salts, triflic anhydride and alkytriflates, CF_3_SO_2_OR. Methyl trfiflate, CF_3_SO_2_OCH_3_, was first synthesised by Gramstadt and Haszeldine by the reaction of silver triflate with methyl iodide [23] (Scheme 1).

**Scheme 2:**
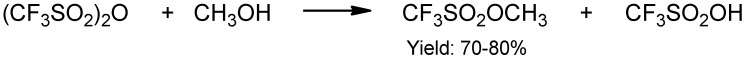
Synthesis of methyl triflate by the reaction of triflic anhydride with methanol [24].

Later on Booth *et al.* [24] used triflic anhydride as a starting material for the synthesis of methyl triflate, CF_3_SO_2_OCH_3_, via reaction with methanol (Scheme 2). 

**Scheme 3:**
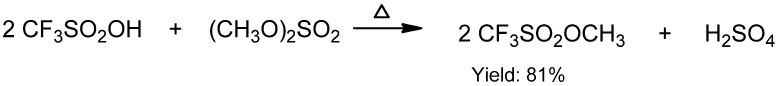
Synthesis of methyl triflate by the reaction of triflic acid with dimethylsulfate [27].

This method can be used to prepare various alkyl triflates, CF_3_SO_2_OR, [25,26], but it is not very economical because half of the triflic anhydride is lost. The yield of methyl triflate in this reaction is also not satisfying. A simpler and less expensive procedure for methyl triflate preparation was developed Beard *et al*. [27] (Scheme 3), but additional efforts are required to utilize the side product, sulphuric acid, contaminated with toxic dimethylsulfate.

## 2. Results and Discussion

**Scheme 4:**
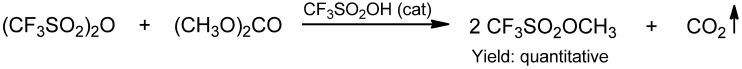
Synthesis of methyl triflate by the reaction of triflic anhydride with dimethylcarbonate [28].

Recently Merck KGaA (Darmstadt, Germany) has developed a new protocol for the preparation of methyl triflate [28] (Scheme 4).

**Scheme 5:**
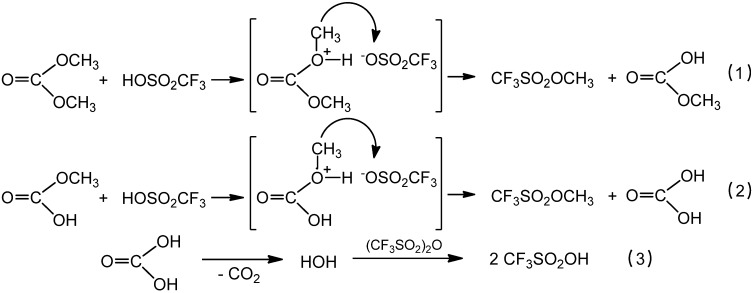
Proposed mechanism of the reaction of triflic anhydride with dimethylcarbonate catalyzed by triflic acid.

Commercial available dimethyl carbonate serves as source for the methyl groups in this reaction. This invented method provides a very convenient access to methyl triflate, CF_3_SO_2_OCH_3_. This chemical is formed in a very good yield and CO_2_ is the only side product in this process. Traces of triflic acid (which is typically present in the commercial triflic anhydride) are enough to catalyse the conversion of triflic acid anhydride and dimethyl carbonate into methyl triflate (Scheme 4). The possible mechanism of this reaction can be described as follows (Scheme 5).

In the first step triflic acid protonates dimethyl carbonate (Equation 1) inducing the methyl cation transfer to the triflate anion resulting in methyl triflate (Equations 1 and 2). Triflic acid is regenerated in the reaction of triflic anhydride with the water which is formed by decomposition of carbonic acid (Scheme 5, Equation 3). According to this mechanism the triflic acid anhydride plays a role as water scavenger. 

**Scheme 6:**
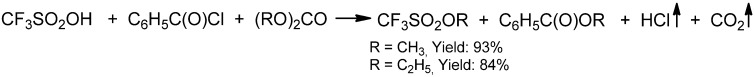
Syntheses of methyl and ethyl triflate by the reaction of triflic acid with dimethyl- and diethyl-carbonate in the presence of benzoyl chloride [29].

The disadvantage of this method is the use of the relatively expensive starting material triflic acid anhydride. To reduce the production costs the combination of triflic acid and acyl or aryl chlorides can be used instead of triflic acid anhydride in this process (Scheme 6) [29]. Acyl or aryl chlorides (for instance cheap and readily available benzoyl chloride) play the role of scavenger of the alcohol and water which are generated as intermediates during the process.

This method provides a very convenient and simple access not only to methyl triflate, but also to ethyl triflate (Scheme 6). Ethyl triflate or methyl triflate can easily be distilled out of the reaction mixture. The residue consists of ethyl or methyl benzoate, which are valuable products as well.

Ethyl triflate and methyl triflate are broadly used in synthetic organic chemistry to alkylate various substrates: *N*-heterocycles [30,31,32,33]; *S*,*N*-heterocycles [34]; organophosphorus compounds [35,36]; imines [37]; enol silyl ethers [38] and aromatic compounds [23,24].

1-Methylimidazole reacts with methyl triflate in dichloromethane yielding 1,3-dimethyl-imidazolium triflate [39]. Bonhöte *et al*. [40] have described the alkylation of 1-alkylimidazoles (Alk = CH_3_, C_2_H_5_, C_4_H_9_) with methyl or ethyl triflate in refluxing 1,1,1-trichloroethane. The yield of triflate ILs was good, but the use of chlorinated solvent was a drawback in this method. 

**Scheme 7:**
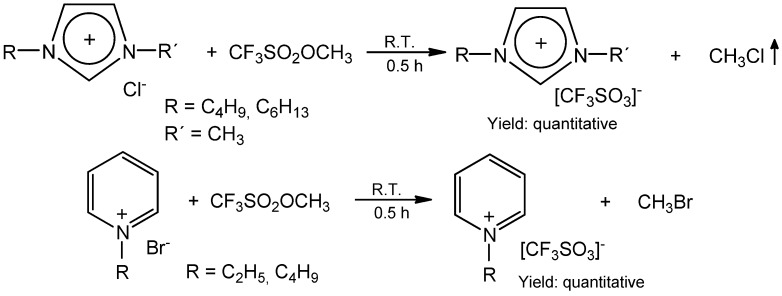
Preparation of triflate ionic liquids from corresponding chlorides or bromides [42,43].

The alkylation ability of methyl triflate is comparable to that of the Meerwein salt [41]. We have used the alkylation power of methyl triflate to convert ionic liquids with chloride or bromide counter-anions to the corresponding ILs with triflate anion (Scheme 7) [42,43].

This is a very convenient method to prepare triflate ionic liquids with various substituents R and R'. The reaction progresses very fast at room temperature under solvent free conditions. The gaseous CH_3_Cl or very volatile CH_3_Br are the only side products in this process. Trimethylsilyl triflate is less active in these reactions, but more convenient to handle [42,43]. The side product, trimethylchlorosilane, can be easily utilised. 

**Scheme 8:**
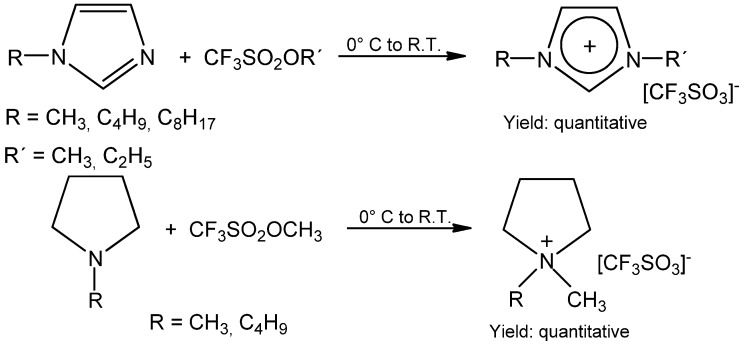
Preparation of triflate ionic liquids by means of alkylation with methyl or ethyl triflate.

To avoid the formation of any side products, the direct alkylation of amines, phosphines or heterocyclic compounds with ethyl or methyl triflate is favored (Scheme 8) [28,29]. This water and halogen free process gives the possibility to prepare triflate ionic liquids of very high quality with residual water below 10 ppm and non-detectable halogenide impurities. This is particularly important for the electrochemical applications of ionic liquids [2].

This method can be applied to the alkylation of trialkylamines, trialkylphosphines, *N*,*N*,*N'*,*N'*-tetra-methylguanidine and *N*,*N*,*N'*,*N'*-tetramethylurea (see Experimental section). The synthesis can be carried out under solvent free conditions at 0 °C. The product yield is close to quantitative. It means that atom economy is very high (close to 100%) and the E-factor has an excellent value [20]. If a solid material is formed during the alkylation process (for example *N*,*N*-dimethylpyrrolidium triflate), diluting the reaction mixture with hexane, which can be easily regenerated, is favored. 

**Scheme 9:**
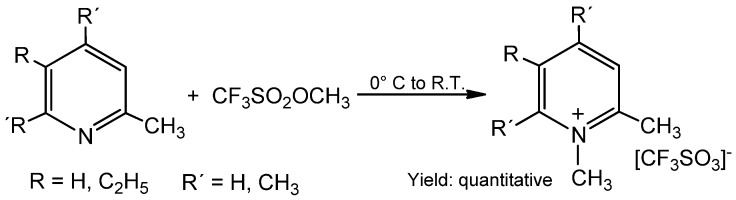
Alkylation of substituted pyridines with methyl triflate.

2-Methyl-5-ethylpyridine and the 2,4,6-trimethylpyridine can be methylated with methyl triflate in quantitative yield (Scheme 9). But, if the steric hindrance is too strong, for example, in the case of 2,4,6-tris(*t*-butyl)pyridine, the reaction proceeds very slowly and the product yield is low (see Experimental section).

**Figure 1:**
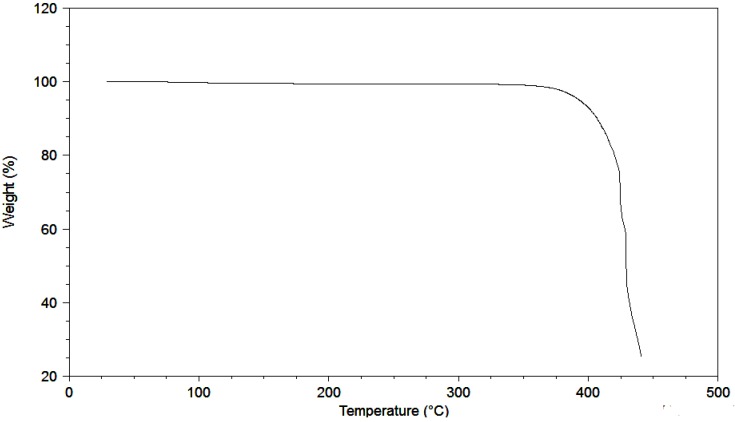
TGA of *N*-butyl-*N*-methylpyrrolidinium trifluoromethanesulfonate. Scan rate: 10 °C/min.

**Figure 2:**
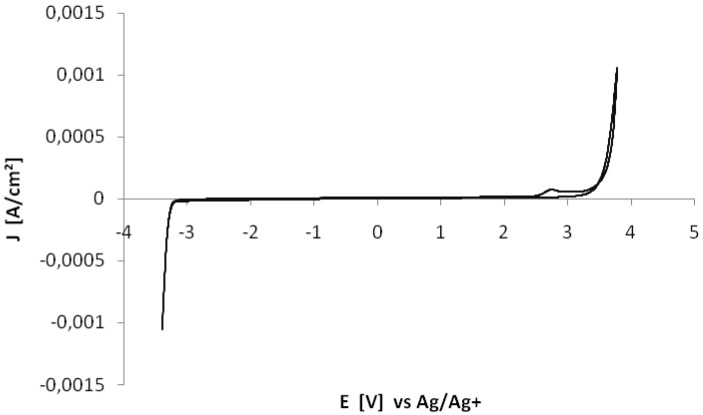
Cyclic voltammogram of *N*-butyl-*N*-methylpyrrolidinium trifluoromethanesulfonate. Scan rate: 20 mV/s.

Ionic liquids with the triflate anion possess high thermal stability (see Figure 1 and Table 1), good electrochemical stability (see Figure 2 and Table 2) and fairly low viscosity (see Table 3 and Table 4). 

**Table 1:** Isothermal TGA measurements at 200 °C and 250 °C.

Ionic Liquid	Weight loss (% per day, 24 h), TGA isothermal mode at 200 °C	Weight loss (% per day, 24 h), TGA isothermal mode at 250 °C
1-Butyl-1-methylpyrrolidinium [CF_3_SO_3_]	0.28	2.8
1-Butyl-3-methylimidazolium [CF_3_SO_3_]	0.55	5.9

**Table 2:** Electrochemical stability of triflate ionic liquids in comparison to the ionic liquids with [(C_2_F_5_)_3_PF_3_] and [BF_4_] anions.

Ionic Liquid	E_(ox)_, V	E_(red)_, V	Window, V
Tetrabutylammonium [(C_2_F_5_)_3_PF_3_]	3.7	−3.3	7.0
Methyl-trioctyl-ammonium [CF_3_SO_3_]	3.4	−3.4	6.8
1-Butyl-1-methylpyrrolidinium [CF_3_SO_3_]	3.4	−3.2	6.6
1-Butyl-3-methylimidazolium [CF_3_SO_3_]	3.0	−2.6	5.6
1-Ethyl-3-methylimidazolium [CF_3_SO_3_]	2.8	−2.5	5.3
1-Ethyl-3-methylimidazolium [BF_4_]	2.6	−2.6	5.2

**Table 3:** Viscosity of 1-ethyl-3-methylimidazolium triflate (EMIM OTF) in comparison to EMIM ionic liquids with alkylsulfate anions, [ROSO_2_O] [36].

Cation	Anion	Dynamic Viscosity, mPa·s			
		20 °C	40 °C	60 °C	80 °C
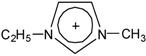	[CH_3_OSO_3_]	84	37	20	12
	[CF_3_SO_3_]	52	26	15	10
	[C_2_H_5_OSO_3_]	117	47	24	14
	[C_4_H_9_OSO_3_]	261	90	40	22

In spite of its higher molecular weight [EMIM][CF_3_SO_3_] is less viscous than [EMIM][CH_3_OSO_3_] (Table 3). The triflate anion is less coordinating than the CH_3_OSO_3_-anion. Weaker interaction between the [CF_3_SO_3_] anion and the EMIM-cation reduces the viscosity of triflate ionic liquids in comparison to methylsulfate ILs [44]. Nevertheless, triflate ILs are more viscous than ionic liquids with weekly coordinating anions such as [(C_2_F_5_)_3_PF_3_] [45] and [(CF_3_SO_2_)_2_N] (Table 4). The viscosity of triflate ionic liquids is influenced by water or organic solvents impurities (decrease of viscosity) and metal triflate impurities (increase of the viscosity value), which can remain in the triflate ILs after metathesis reaction.

**Table 4:** Viscosity and density of 1-hexyl-3-methylimidazolium triflate in comparison to ionic liquids with other anions.

Ionic Liquid	Kinematic Viscosity (20 °C), mm^2^/s	Density (20 °C), g/cm^3^	Dynamic Viscosity (20 °C), mPa·s
1-Hexyl-3-methylimidazolium Chloride	7453	1.05	7826
1-Hexyl-3-methylimidazolium [PF_6_]	548	1.30	712
1-Hexyl-3-methylimidazolium [BF_4_]	195	1.15	224
1-Hexyl-3-methylimidazolium [CF_3_SO_3_]	160	1.24	198
1-Hexyl-3-methylimidazolium [(C_2_F_5_)_3_PF_3_]	74	1.56	115
1-Hexyl-3-methylimidazolium [(CF_3_SO_2_)_2_N]	65	1.38	90

The possible applications of triflate ionic liquids themselves or in combination with Brønsted acids for dehydration of alcohols, deprotection of ketals or synthesis of chromane derivatives are described in the cited patents applications [46,47,48].

## 3. Experimental

### 3.1. Chemicals

Commercially available chemicals have been used. Triflic acid and triflic acid anhydride was provided by Merck KGaA (Darmstadt, Germany) company and used without further purification. Methyl trifluoromethanesulfonate (methyl triflate) and ethyl trifluoromethanesulfonate (ethyl triflate) were obtained and distilled as described below and stored under a nitrogen atmosphere. Heterocyclic bases were distilled over CaH_2_ and stored under a nitrogen atmosphere. 

### 3.2. Analytical Procedures

#### 3.2.1. NMR Spectroscopy

NMR samples were measured in 5 mm precision glass NMR tube (Wilmad 507 or 528 PPT) at 24 °C. NMR spectra were recorded in the deuterium-locked mode on a Bruker Avance III spectrometer equipped with a 9.3980 T cryomagnet. The ^1^H- and ^19^F-NMR spectra were acquired using a 5 mm ^1^H/^19^F combination probe operating at 400.17 and 376.54 MHz respectively. ^13^C-NMR spectra were obtained using a 5 mm broad-band inverse probe operating at 100.61 MHz. Spectra were recorded using various memory sizes, optimal acquisition times, and relaxation delays (0.5–2 s). The ^1^H-NMR chemical shifts were referenced with respect to tetramethylsilane (TMS) using the chemical shifts for the solvents CH_2_Cl_2_ (5.32 ppm) and CH_3_CN (1.95 ppm). The ^13^C-NMR chemical shifts were referenced with respect to TMS using the chemical shifts for the solvents CH_2_Cl_2_ (53.5 ppm) and CH_3_CN (118.7 ppm). The ^19^F-NMR spectra were referenced with respect to CFCl_3_ using either the internal standards C_6_F_6_ (−162.9 ppm) or C_6_H_5_CF_3_ (−63.9 ppm) or externally to a neat CFCl_3_ reference sample at 24 °C. 

#### 3.2.2. Elemental Analysis

Elemental analysis was performed using a HEKATECH EA 3000 elemental analyser (HEKAtech GmbH, Wegberg, Germany) with Callidus software.

#### 3.2.3. Thermal Analyses

The thermal analyses were performed using a NETZSCH 409C STA (Selb, Germany).

#### 3.2.4. Viscosity

Viscosity and density of ionic liquids were measured on the viscosimeter SVM 3000 (Anton Paar, Graz, Austria). 

#### 3.2.5. Cyclic Voltammetry

For electrochemical stability measurements an Autolab PGSTAT 30 (Metrohm Autolab B.V., Utrecht, The Netherlands) was used. Cyclic voltammograms were recorded for 0.5 M solutions in CH_3_CN at glassy carbon (gc) working electrode; auxiliary electrode was Pt and Ag/AgNO_3_ (CH_3_CN) was used as reference electrode. The purity (quality) of triflate ionic liquids was proven by measuring of residual water (Karl-Fischer titration; 831 KF-Coulometer, Metrohm, Filderstadt, Germany) and chloride or bromide (ion-chromotography, Metrohm Advanced IC System; stationary phase: Metrosep A SUPP4-150). Contents of residual water and halides were below 100 ppm.

### 3.3. Syntheses

#### *Methyl Trifluoromethanesulfonate* (CF_3_SO_2_OCH_3_)

Method (**a**): Trifluoromethanesulfonic acid anhydride (2,300 g, 8.15 mol) was slowly added to freshly distilled dimethyl carbonate (808 g, 8.97 mol) under vigorous stirring at 25 °C. The solution was heated (approximately at 70 °C) until a moderate stream of CO_2_ runs through the H_2_SO_4_-filled bubbler. Stirring was continued for 48 h at 70 °C. The excess of dimethyl carbonate (b.p. 90 °C) was distilled off over a short column under a dry atmosphere. The yield of the colourless raw product is close to 100%. Purification by fractionated distillation gives 2,515 g (15.33 mol, 94% yield) of methyl trifluoromethanesulfonate (b.p. 94–96 °C); ^1^H-NMR (CD_3_CN) δ, ppm: 3.75 s; ^19^F-NMR (CD_3_CN) δ, ppm: −76.3 s.

Method (**b**): Trifluoromethanesulfonic acid (3,000 g, 19.99 mol) was slowly added to freshly distilled benzoyl chloride (2,810 g, 19.99 mol) with mechanical stirring under a very slight positive N_2_ pressure. After the addition was completed (45 min), the solution was heated to 80 °C. Freshly distilled dimethyl carbonate (1,715 g, 19.04 mol) was dropped slowly into the solution under vigorous stirring over a period of 7 h. During the addition a stream of CO_2_ and HCl passed through the H_2_SO_4_-filled bubbler. Stirring was continued for 4 h at 85 °C to complete the reaction. The raw product was distilled off with a short column under a dry atmosphere until the head-temperature increased above 115 °C. The yield of the colourless raw product is close to 100%. Purification by fractional distillation gives 2,906 g (17.12 mol, 93% yield) of methyl trifluoromethanesulfonate (b.p. 94–96 °C); ^1^H-NMR (CD_3_CN), δ, ppm: 3.76 s; ^19^F-NMR (CD_3_CN), δ, ppm: δ −76.3 s.

#### *Ethyl Trifluoromethanesulfonate* (CF_3_SO_2_OC_2_H_5_)

Trifluoromethanesulfonic acid (918 g, 6.12 mol) was slowly added to freshly distilled benzoyl chloride (860 g, 6.12 mol) under a very slight positive N_2_ pressure with mechanical stirring. After the addition was completed (20 min), the solution was heated to 95 °C. Freshly distilled diethyl carbonate (690 g, 5.83 mol) was then dropped slowly into the solution under vigorous stirring over a period of 3 h. During the addition a gas stream of of CO_2_ and HCl passed through the H_2_SO_4_-filled bubbler. Stirring was continued for 1 h at 100 °C to complete the reaction. The raw product was distilled off immediately over a short column under a dry atmosphere until the head-temperature raised above 125 °C. The yield of the colourless raw product was 92%. Purification by fractional distillation gives 874 g (4.91 mol, 84%) of ethyl trifluoromethanesulfonate (b. p. 112–114 °C); ^1^H-NMR (CD_3_CN), δ, ppm: 1.03 t (3H, CH_3_, ^3^*J*_H,H_* = * 7.2 Hz), 4.19 q (2H, CH_2_, ^3^*J*_H,H_* = * 7.2 Hz); ^19^F-NMR (CD_3_CN), δ, ppm: −76.4 s.

#### *1,3-Dimethylimidazolium Trifluoromethanesulfonate* [C_5_H_9_N_2_][CF_3_SO_3_]

Freshly distilled methyl trifluoromethanesulfonate (1,486 g, 9.06 mol) was dropped slowly into a mechanically stirred solution of *N*-methylimidazole (730 g, 8.89 mol) in hexane (1,000 mL) over a period of 16 h (1,300 g were dropped in at 0 °C and 159 g at 25 °C). Stirring (700 rpm) was continued for 12 h at 25 °C to complete the reaction. During this time the product precipitated as a colourless solid. The solvent and the excess of methyl trifluoromethanesulfonate (27 g, 0.17 mol) were decanted and finally the product was evaporated at 0.01 mbar and 25 °C for 6 h. 1,3-Dimethylimidazolium-trifluoromethanesulfonate was obtained as a colourless solid (2,189 g, 8.89 mol, quantitative yield, m.p.: 56 °C); ^1^H-NMR (CD_3_CN), δ, ppm: 3.85 s (6H, 2CH_3_), 7.37 s (2H), 8.52 s (1H); ^19^F-NMR (CD_3_CN), δ, ppm: −79.3 s (3F, CF_3_S); ^13^C-NMR (CD_3_CN), δ, ppm: 35.4 q, 120.8 q (CF_3_S), 123.2 d, m, 136.5 d, m; Anal. Calcd. for C_6_H_9_O_3_N_2_F_3_S: C, 29.27; H, 3.68; N, 11.38; S, 13.02. Found: C, 30.03; H, 4.10; N, 11.57; S, 13.50.

#### *1-Ethyl-3-methylimidazolium Trifluoromethanesulfonate* [C_6_H_1__1_N_2_][CF_3_SO_3_]

Freshly distilled ethyl trifluoromethanesulfonate (895 g, 5.02 mol) was dropped slowly into magnetically stirred *N*-methylimidazole (400 g, 4.87 mol) at 0 °C over a period of 6 h. Stirring (1,000 rpm) was continued for 12 h at 30 °C to complete the reaction. Finally the excess of ethyl trifluoromethanesulfonate (27 g, 0.15 mol) was evaporated at 0.01 mbar and 25 °C. The product (1,268 g, 4.87 mol, quantitative yield) was obtained as a clear and colourless liquid; ^1^H-NMR (CD_3_CN), δ, ppm: 1.44 t (3H, CH_3_, ^3^*J*_H,H_* = * 7.3 Hz), 3.86 s (3H, CH_3_), 4.20 q (2H, CH_2_, ^3^*J*_H,H_* = * 7.3 Hz), 7.44 s (1H), 7.50 s (1H), 8.70 s (1H); ^19^F-NMR (CD_3_CN), δ, ppm: −79.3 s (3F, CF_3_S); ^13^C-NMR (CD_3_CN), δ, ppm: 14.3 q, 35.4 q, 44.5 t, 120.8 q (CF_3_S), 121.7 d,m, 123.3 d,m, 135.9 d,m; Anal. Calcd. for C_7_H_11_O_3_N_2_F_3_S: C, 32.31; H, 4.26; N, 10.76; S, 12.32. Found: C, 32.47; H, 4.50; N, 10.86; S, 12.39.

#### *1-Butyl-3-methylimidazolium Trifluoromethanesulfonate* [C_8_H_1__5_N_2_][CF_3_SO_3_]

Method (**a**): Freshly distilled methyl trifluoromethanesulfonate (1,341 g, 8.17 mol) was dropped slowly into magnetically stirred *N*-butylimidazole (1,000 g, 8.05 mol) during 14 h at 0 °C. Stirring (1,000 rpm) was continued for 3 h at 25 °C to complete the reaction. Finally the excess of methyl trifluoromethanesulfonate (20 g, 0.12 mol) was evaporated at 0.01 mbar and 25 °C within 10 h. The product (2,321 g, 8.05 mol, quantitative yield) was obtained as a clear and nearly colourless liquid; ^1^H-NMR (CD_3_CN), δ, ppm: 0.93 t (3H, CH_3_, ^3^*J*_H,H_* = * 7.3 Hz), 1.33 m (2H, CH_2_), 1.82 m (2H, CH_2_), 3.86 s (3H, CH_3_), 4.17 q (2H, CH_2_, ^3^*J*_H,H_* = * 7.3 Hz), 7.42 s (1H), 7.47 s (1H), 8.68 s (1H); ^19^F-NMR (CD_3_CN), δ, ppm: −79.3 s (3F, CF_3_S); ^13^C-NMR (CD_3_CN), δ, ppm: 12.4 q, 18.6 t, 31.3 t, 35.5 q, 48.9 t, 120.8 q (CF_3_S), 122.0 d,m, 123.3 d,m, 136.0 d,m; Anal. Calcd. for C_9_H_15_O_3_N_2_F_3_S: C, 37.50; H, 5.24; N, 9.72; S, 11.12. Found: C, 37.30; H, 5.39; N, 9.60; S, 10.53.

Method (**b**): A mixture of 1-butyl-3-methylimidazolium chloride (1.04 g, 5.95 mmol) and methyl triflate (CF_3_SO_2_OCH_3_, 1.67 g, 10.2 mmol) was stirred at room temperature for 30 min. The residue was dried for 30 min in vacuum at 0.13 mbar and 120 °C (oil-bath temperature), giving 1.71 g (a quantitative yield) of 1-butyl-3-methylimidazolium triflate. The product was characterised by NMR spectroscopy. The spectra are identical to those of the product obtained by Method (**a**).

Method (**c**): A mixture of 1-butyl-3-methylimidazolium chloride (1.67 g, 9.56 mmol) and trimethylsilyl triflate ([CF_3_SO_2_OSi(CH_3_)_3_, 2.36 g, 10.6 mmol] was stirred at room temperature for 30 min. The residue was dried for 30 min in vacuum at 0.13 mbar and 120 °C (oil-bath temperature), giving 2.75 g (a quantitative yield) of 1-butyl-3-methylimidazolium triflate. The product was characterised by means of NMR spectroscopy. The spectra are identical to those of the product obtained by Method (**a**).

#### *1-Hexyl-3-methylimidazolium-trifluoromethanesulfonate* [C_1__0_H_1__9_N_2_][CF_3_SO_3_]

Method (**a**): A mixture of 1-hexyl-3-methylimidazolium chloride (1.80 g, 8.88 mmol) and methyl triflate (CF_3_SO_2_OCH_3_, 1.52 g, 9.26 mmol) was stirred at room temperature for 30 min. NMR measurements show the completeness of the reaction. The residue was dried for 30 min under vacuum at 0.13 mbar and 120 °C (oil-bath temperature), giving 2.80 g (a quantitative yield) of 1-hexyl-3-methylimidazolium triflate; ^1^H-NMR (CD_3_CN), δ, ppm: 0.87 m (3H, CH_3_), 1.29 m (6H, 3CH_2_), 1.81 m (2H, CH_2_), 3.82 s (3H, CH_3_), 4.11 t (2H, CH_2_), 7.35 d,d (1H, CH), 7.39 d,d (1H, CH), 8.55 br.s (1H, CH), ^3^*J*_H,H_ = 6.9 Hz, *J*_H,H_ = 1.5 Hz; ^19^F-NMR (CD_3_CN), δ, ppm: −78.1 s (3F, SCF_3_).

Method (**b**): A mixture of 1-hexyl-3-methylimidazolium chloride (1.81 g, 8.93 mmol) and trimethylsilyl triflate [CF_3_SO_2_OSi(CH_3_)_3_, 2.62 g, 9.26 mmol] was stirred at room temperature for 4 h. The residue was dried for 30 min under vacuum at 0.13 mbar and 120 °C (oil-bath temperature), giving 2.81 g (a quantitative yield) of 1-hexyl-3-methylimidazolium triflate. The product was characterised by means of NMR spectroscopy. The spectra are identical to those of the product obtained by Method (**a**).

#### *3-Octyl-1-methylimidazolium-trifluoromethanesulfonate* [C_1__2_H_2__3_N_2_][CF_3_SO_3_]

Freshly distilled methyl trifluoromethanesulfonate (1,010 g, 6.15 mol) was dropped slowly into magnetically stirred *N*-octylimidazole (1,100 g, 6.10 mol) over a period of 10 h at 0 °C. Stirring (1,000 rpm) was continued for 2 h at 20 °C and 1 hour at 40 °C to complete the reaction. Finally the excess of methyl trifluoromethanesulfonate (9 g, 0.05 mol) was evaporated at 0.01 mbar and 25 °C within 10 h. The product (2101 g, 6.10 mol, quantitative yield) was obtained as a clear and nearly colourless liquid; ^1^H-NMR (CD_3_CN), δ, ppm: 0.89 t (3H, CH_3_, ^3^*J*_H,H_* = * 7.0 Hz), 1.30 m (10H, 5CH_2_), 1.84 m (2H, CH_2_), 3.86 s (3H, CH_3_), 4.15 t (2H, CH_2_, ^3^*J*_H,H_* = * 7.3 Hz), 7.41 s (1H), 7.46 s (1H), 8.63 s (1H); ^19^F-NMR (CD_3_CN), δ, ppm: −79.3 s (3F, CF_3_S); ^13^C-NMR (CD_3_CN), δ, ppm: 14.7 q, 23.7 t, 27.0 t, 29.9 t, 30.1 t, 31.0 t, 32.8 t, 37.1 q, 50.8 t, 120.8 q (CF_3_S), 123.6 d, m, 125.0 d, m, 137.5 d, m; Anal. Calcd. for C_13_H_23_O_3_N_2_F_3_S: C, 45.34; H, 6.73; N, 8.13; S, 9.31. Found: C, 45.29; H, 6.78; N, 7.77; S, 8.61.

#### *1-Butyl-3-ethylimidazolium-trifluoromethanesulfonate* [C_9_H_1__7_N_2_][CF_3_SO_3_]

Freshly distilled ethyl trifluoromethanesulfonate (1,240 g, 6.96 mol) was dropped slowly into magnetically stirred *N*-butylimidazole (840 g, 6.76 mol) during 9 h at 0 °C. Stirring (1,000 rpm) was continued for 12 h at 30 °C to complete the reaction. Finally the excess of ethyl trifluoromethanesulfonate (35 g, 0.20 mol) was evaporated at 0.01 mbar and 25 °C. The product (2,045 g, 6.76 mol, quantitative yield) was obtained as a clear and colourless liquid; ^1^H-NMR (CD_3_CN), δ, ppm: 0.94 t (3H, CH_3_, ^3^*J*_H,H_* = * 7.3 Hz), 1.34 m (2H, CH_2_), 1.48 t (3H, CH_3_, ^3^*J*_H,H_* = * 7.3 Hz), 1.83 m (2H, CH_2_), 4.17 t (2H, CH_2_, ^3^*J*_H,H_* = * 7.3 Hz), 4.21 q (2H, CH_2_, ^3^*J*_H,H_* = * 7.3 Hz ), 7.48 s (1H), 7.49 s (1H), 8.73 s (1H); ^19^F-NMR (CD_3_CN), δ, ppm: −79.3 s (3F, CF_3_S); ^13^C-NMR (CD_3_CN), δ, ppm: 12.4 q, 14.2 q, 31.3 t, 44.5 t, 49.0 t, 120.8 q (CF_3_S), 121.8 d,m, 122.4 d,m, 135.0 d,m; Anal. Calcd. for C_10_H_17_O_3_N_2_F_3_S: C, 39.73; H, 5.67; N, 9.27; S, 10.61. Found: C, 39.13; H, 5.43; N, 9.32; S, 10.63.

#### *N,N-Dimethylpyrrolidinium Trifluoromethanesulfonate* [C_7_H_1__4_N][CF_3_SO_3_]

Freshly distilled methyl trifluoromethanesulfonate (272 g, 1.66 mol) was added slowly (within 45 min) to the stirred solution of *N*-methylpyrrolidine (141.1 g, 1.66 mol) in dry hexane (800 mL) at 0 °C. After that the reaction mixture was heated and kept reflux for 15 min (oil-bath temperature is 70–75 °C). After cooling to room temperature a white precipitate was filtered off, washed twice with hexane (100 mL) and dried under vacuum (0.3 mbar) at 110 °C for 3 h. The *N*,*N*-dimethylpyrrolidinium trifluoromethanesulfonate was obtained as a white solid material (409 g, 99%); ^1^H-NMR (CD_3_CN), δ, ppm: 2.17 m (4H, 2CH_2_), 3.07 s (6H, 2CH_3_), 3.45 m (4H, 2CH_2_); ^19^F-NMR (CD_3_CN), δ, ppm: −78.0 s (3F, CF_3_S); Anal. Calcd. for C_7_H_14_F_3_NO_3_S: C, 33.73; H, 5.66; N, 5.62; S, 12.86. Found: C, 33.66; H, 5.68; N, 5.60; S, 13.06.

#### *N-Butyl-N-methylpyrrolidinium Trifluoromethanesulfonate* [C_9_H_2__0_N][CF_3_SO_3_]

Freshly distilled methyl trifluoromethanesulfonate (990 g, 6.03 mol) was dropped slowly into magnetically stirred *N*-butylpyrrolidine (764 g, 6.00 mol) over a period of 24 h at 0 °C. Stirring (1,000 rpm) was continued for 3 h at 25 °C to complete the reaction. Finally the excess of methyl trifluoromethanesulfonate (5 g, 0.03 mol) was evaporated at 0.01 mbar and 25 °C within 10 h. The product (1,749 g, 6.00 mol, quantitative yield) was obtained as a clear and nearly colourless liquid; ^1^H-NMR (CD_3_CN), δ, ppm: 0.97 t (3H, CH_3_, ^3^*J*_H,H_* = * 7.4 Hz), 1.38 m (2H, CH_2_), 1.73 m (2H, CH_2_), 2.16 m (4H, 2CH_2_), 2.98 s (3H, CH_3_), 3.28 m (2H, CH_2_), 3.45 m (4H, CH_2_); ^19^F-NMR (CD_3_CN), δ, ppm: −79.2 s (3F, CF_3_S); ^13^C-NMR (CD_3_CN), δ, ppm: 12.5 q, 19.1 t, 21.0 t, 25.0 t, 47.8 q, 63.6 t, 63.9 t, 120.8 q (CF_3_S) ; Anal. Calcd. for C_10_H_20_O_3_NF_3_S: C, 41.23; H, 6.92; N, 4.81; S, 11.01. Found: C, 40.85; H, 6.81; N, 4.96; S, 10.42.

#### *N,N-Dimethylpiperidinium Trifluoromethanesulfonate* [C_8_H_1__6_N][CF_3_SO_3_]

Freshly distilled methyl trifluoromethanesulfonate (12.83 g, 78.2 mmol) was added slowly (within 45 min) to the stirred solution of *N*-methylpiperidine (7.76 g, 78.2 mol) in dry hexane (150 mL) at room temperature. The warm reaction mixture was left stirring for 30 min and then cooled down to room temperature. The white precipitate was filtered off, washed twice with hexane (10 mL) and dried under 0.3 mbar vacuum at 80 °C to give a white solid material (19.72 g, 96%). The melting point after crystallisation from methanol was 255–256 °C; ^1^H-NMR (CD_3_CN), δ, ppm: 1.79 m (6H, 3CH_2_), 3.02 s (6H, 2CH_3_), 3.27 m (4H, 2CH_2_); ^19^F-NMR (CD_3_CN), δ, ppm: −78.1 s (3F, CF_3_S); Anal. Calcd. for C_8_H_16_F_3_NO_3_S: C, 36.50; H, 6.13; N, 5.32; S, 12.18. Found: C, 33.44; H, 6.07; N, 5.31; S, 12.20.

#### *N-Methyl-3-butylpyridinium Trifluoromethanesulfonate* [C_9_H_2__0_N][CF_3_SO_3_]

Freshly distilled methyl trifluoromethanesulfonate (23.00 g, 0.14 mol) was dropped slowly into magnetically stirred 3-butylpyridine (18.13 g, 0.13 mol) over a 2 hour period at 0 °C. Stirring (800–1,000 rpm) was continued for 1 h at 25 °C to complete the reaction. Finally the excess of methyl trifluoromethanesulfonate (1.00 g, 0.01 mol) was evaporated at 0.01 mbar and 25 °C within 5 h. The title compound (40.13 g, 0.13 mol, quantitative yield) was obtained as a clear and pale yellow liquid; ^1^H-NMR (CD_3_CN), δ, ppm: 0.90 t (3H, CH_3_, ^3^*J*_H,H_* = * 7.3 Hz), 1.34 m (2H, CH_2_), 1.64 m (2H, CH_2_), 2.82 t (2H, CH_2_, ^3^*J*_H,H_* = * 7.8 Hz,), 4.37 s (3H, CH_3_), 7.93 m (1H, CH), 8.40 m (1H, CH), 8.67 m (1H, CH), 8.75 s (1H, CH); ^19^F-NMR (CD_3_CN), δ, ppm: −77.9 s (3F, CF_3_S).

#### *N-Ethylpyridinium Trifluoromethanesulfonate* [C_7_H_1__0_N][CF_3_SO_3_]

Method (**a**): A mixture of *N*-ethylpyridinium bromide (1.56 g, 8.29 mmol) and methyl triflate (CF_3_SO_2_OCH_3_, 1.67 g, 10.2 mmol) was stirred at room temperature for 30 min. The residue was dried for 30 min under vacuum (0.13 mbar) at 120 °C (oil-bath temperature), giving 2.13 g (a quantitative yield) of *N*-ethylpyridinium triflate; ^1^H-NMR (CD_3_CN), δ, ppm: 1.58 t (3H, CH_3_), 4.59 q (2H, CH_2_), 8.02 m (2H, 2CH), 8.49 t,t (1H, CH), 8.78 d (2H, 2CH), ^3^*J*_H,H_ = 7.3 Hz, ^3^*J*_H,H_ = 7.6 Hz, ^3^*J*_H,H_ = 6.1 Hz, ^4^*J*_H,H_ = 1.2 Hz; ^19^F-NMR (CD_3_CN), δ, ppm: −78.0 s (3F, CF_3_).

Method (**b**): A mixture of *N*-ethylpyridinium bromide (1.85 g, 9.84 mmol) and trimethylsilyl triflate [CF_3_SO_2_OSi(CH_3_)_3_, 2.54 g, 11.4 mmol) was stirred at room temperature for 4 h. The residue was dried for 30 min under vacuum (0.13 mbar) at 120 °C (oil-bath temperature), giving 2.53 g (a quantitative yield) of *N*-ethylpyridinium triflate. The product was characterised by means of NMR spectroscopy. The spectra are identical to those of the product obtained using Method (**a**). 

#### *N-Butylpyridinium Trifluoromethanesulfonate* [C_9_H_1__4_N][CF_3_SO_3_]

Method (**a**): A mixture of *N*-butylpyridinium bromide (4.31 g, 19.9 mmol) and of methyl triflate (CF_3_SO_2_OCH_3_, 4.41 g, 26.9 mmol) was stirred at room temperature for 30 min. The residue was dried for 30 min under vacuum (0.13 mbar) at 120 °C (oil-bath temperature), giving 5.68 g (a quantitative yield) of *N*-butylpyridinium triflate; ^1^H-NMR (CD_3_CN), δ, ppm: 0.93 t (3H, CH_3_), 1.35 m (2H, CH_2_), 1.93 m (2H, CH_2_), 4.53 t (2H, CH_2_), 8.02 m (2H, 2CH), 8.50 t (1H, CH), 8.74 d (2H, 2CH), ^3^*J*_H,H_ = 7.4 Hz, ^3^*J*_H,H_ = 7.6 Hz, ^3^*J*_H,H_ = 7.9 Hz, ^3^*J*_H,H_ = 6.1 Hz; ^19^F-NMR (CD_3_CN), δ, ppm: −78.0 s (3F, CF_3_).

Method (**b**): A mixture of *N*-butylpyridinium bromide (1.42 g, 6.57 mmol) and of trimethylsilyl triflate [CF_3_SO_2_OSi(CH_3_)_3_, 1.73 g, 7.78 mmol) was stirred at room temperature for 4 h. The residue was dried for 30 min under vacuum (0.13 mbar) at 120 °C (oil-bath temperature), giving 1.87 g (a quantitative yield) of *N*-butylpyridinium triflate. The product was characterised by means of NMR spectroscopy. The spectra are identical to those described in the experiment (**a**).

#### 1,2-Dimethyl-5-ethylpyridinium-trifluoromethanesulfonate

Freshly distilled methyl trifluoromethanesulfonate (45.0 g, 0.27 mol) was dropped slowly into magnetically stirred 5-ethyl-2-methylpyridine (32.0 g, 0.26 mol) over a period of 4 h at 0 °C. Stirring (1,000 rpm) was continued for 3 h at 25 °C to complete the reaction. Finally the excess of methyl trifluoromethanesulfonate (1.7 g, 0.01 mol) was evaporated at 0.01 mbar and 25 °C within 10 h. The product (75.3 g, 0.26 mol, quantitative yield) was obtained as a clear and nearly colourless liquid; ^1^H-NMR (CD_3_CN), δ, ppm: 1.29 t (3H, ^3^*J*_H,H_* = * 7.6 Hz, CH_2_CH_3_), 2.73 s (3H, CH_3_), 2.81 t (2H, ^3^*J*_H,H_* = * 7.6 Hz , CH_2_CH_3_), 4.17 s (3H, CH_3_), 7.80 d (1H, *J*_H,H_* = * 8.2), 8.24 d (1H, *J*_H,H_* = * 8.2), 8.56 s (1H). ^19^F-NMR (CD_3_CN), δ, ppm: −79.2 s (3F, CF_3_S); ^13^C-NMR (CD_3_CN), δ, ppm: 13.7 q , 19.2 q, 24.9 t, 45.5 q, 121.1 q (CF_3_S), 129.0 d, 143.1 d, 144.8 d, 145.8 s, 153.3 s Anal. Calcd. for C_10_H_14_O_3_NF_3_S: C, 42.10; H, 4.95; N, 4.91; S, 11.24. Found: C, 41.55; H, 4.83; N, 4.90; S, 11.40.

#### *1,2,4,6-Tetramethylpyridinium Trifluoromethanesulfonate* [C_9_H_1__4_N][CF_3_SO_3_]

Freshly distilled methyl trifluoromethanesulfonate (15.0 g, 0.09 mol) was dropped slowly into a magnetically stirred solution of 2,4,6-trimethylpyridine (10.0 g, 0.08 mol) in hexane (100 mL) over a period of 2 h at 25 °C. With the first drops a colourless solid material precipitated. Stirring (1,200 rpm) was continued for 1 h at 25 °C to complete the reaction. The deposit was isolated by filtration and washed twice with fresh hexane. Finally the product was evaporated at 0.01 mbar and 25 °C for 2 h. 1,2,4,6-Tetramethylpyridinium trifluoromethanesulfonate (23.5 g, 0.08 mol, quantitative yield) was obtained as a colourless solid (m.p.: 145 °C); ^1^H-NMR (CD_3_CN), δ, ppm: 2.51 s (3H, CH_3_), 2.71 s (6H, 2CH_3_), 3.94 s (3H, CH_3_), 7.56 s (2H, 2CH); ^19^F-NMR (376.54 MHz, CD_3_CN), δ, ppm: −79.3 s (3F, CF_3_S); ^13^C-NMR (CD_3_CN), δ, ppm: 21.8 q, 22.2 q, 40.9 q, 122.5 q (CF_3_S), 129.1 d, 156.3 s, 159.1 s; Anal. Calcd. for C_10_H_14_O_3_NF_3_S: C, 42.10; H, 4.95; N, 4.91; S, 11.24. Found: C, 42.59; H, 5.17; N, 4.98; S, 11.68.

#### *1-Methyl-2,4,6,-tris(t-butyl)pyridnium Trifluoromethanesulfonate* [C_1__8_H_3__2_N][CF_3_SO_3_]

Freshly distilled methyl trifluoromethanesulfonate (10.0 g, 60 mmol) was added to solid 2,4,6-tris(*t*-butyl)pyridine (1.0 g, 4 mmol). The suspension was heated to reflux (bath 100 °C) and forms a solution at 70 °C. After 8 h of stirring the solution was cooled down to room temperature (a suspension is formed again at 70 °C). The raw solid material contains only 6 % of the expected product. After two recrystallizations from dichloromethane/hexane (1/10, vol/vol), the colourless solid product obtained was evaporated at 0.01 mbar and 25 °C within 8 h to give the product (0.07 g, 0.2 mmol, 4% yield) as a colourless solid (m.p.: 186 °C); ^1^H-NMR (CD_3_CN), δ, ppm: 1,55 s (9H, 3CH_3_), 1.66 s (18H, 6CH_3_), 4.36 s (3H, NCH_3_), 7.92 s (2H, 2CH); ^19^F-NMR (CD_3_CN), δ, ppm: −79.3 s (3F, CF_3_S); Anal. Calcd. for C_19_H_32_O_3_NF_3_S: C, 55.45; H, 7.84; N, 3.40; S, 7.79. Found: C, 54.11; H, 7.80; N, 3.69; S, 7.75.

#### *Methyl trioctylammonium Trifluoromethanesulfonate* [(C_8_H_1__7_)_3_NCH_3_][CF_3_SO_3_]

Freshly distilled methyl trifluoromethanesulfonate (715 g, 4.36 mol) was dropped slowly into a magnetically stirred solution of trioctylamine (1,526 g, 4.31 mol) in dichloromethane (700 mL) within 24 h at 0 °C. Stirring (1,000 rpm) was continued for 3 h at 25 °C to complete the reaction. Dichloromethane (b.p. 40 °C) was distilled of under atmospheric pressure. Residual dichloromethane and the excess of methyl trifluoromethanesulfonate (7 g, 0.05 mol) were evaporated at 0.01 mbar within 14 h. During the evaporation the temperature was increased slowly to 65 °C to avoid the crystallization of the product. Methyl trioctylammonium trifluoromethanesulfonate (2,234 g, 4.31 mol, quantitative yield) was obtained as a colourless solid (m.p.: 52 °C); ^1^H-NMR (CD_3_CN), δ, ppm: 0.92 t (9H, 3CH_3_, ^3^*J*_H,H_* = * 6.7 Hz), 1.34 m (30H, 15CH_2_), 1.67 m (6H, 3CH_2_), 2.92 s (3H, NCH_3_), 3.16 m (6H, 3CH_2_); ^19^F-NMR (CD_3_CN), δ, ppm: −79.1 (s, 3F, CF_3_S); ^13^C-NMR (CD_3_CN), δ, ppm: 13.1 q, 21.4 t, 22.0 t, 25.5 t, 28.3 t, 28.4 t, 31.1 t, 47.6 q, 61.2 t, 120.9 q (CF_3_S); Anal. Calcd. for C_26_H_54_O_3_NF_3_S: C, 60.31; H, 10.51; N, 2.71; S, 6.19. Found: C, 59.82; H, 10.51; N, 2.76; S, 6.26.

#### *Methyl triethylphosphonium Trifluoromethanesulfonate* [(C_2_H_5_)_3_PCH_3_][CF_3_SO_3_]

Freshly distilled methyl trifluoromethanesulfonate (11.19 g, 68.2 mmol) was added slowly (within 10 min) to a stirred solution of triethylphosphine (8.05 g, 68.2 mmol) in dry hexane (150 mL) at room temperature. The warm reaction mixture was left stirring for 30 min and cooled down to room temperature. The white precipitate was filtered off, washed twice with hexane (10 mL) and dried under vacuum (0.3 mbar) at 60 °C to give the title compound (19.02 g, 99% yield) as a white solid material; ^1^H-NMR (acetone-d_6_): δ, ppm: 1.30 d,t, (9H, 3CH_3_^3^*J*_H,H_ = 7.7 Hz, ^3^*J*_P,H_ = 18.8 Hz), 1.95 d (3H, PCH_3_, ^2^*J*_P,H_ = 13.7 Hz), 2.40 d,q (6H, 3CH_2_, ^2^*J*_P,H_ = 13.8 Hz); ^19^F-NMR (CD_3_CN), δ, ppm: −77.8 s (3F, CF_3_S); Anal. Calcd. for C_8_H_18_F_3_O_3_PS: C, 34.04; H, 6.43; S, 11.36. Found: C, 33.72; H, 6.57; S, 11.14.

#### *Trihexyl tetradecylphosphonium Trifluoromethanesulfonate* [(C_6_H_1__3_)_3_PC_1__4_H_2__9_][CF_3_SO_3_]

Method (**a**): A mixture of trihexyl tetradecylphosphonium chloride (1.75 g, 3.37 mmol) and methyl triflate (CF_3_SO_2_OCH_3_, 2.59 g, 15.8 mmol) was stirred at room temperature for 30 min. The residue was dried for 30 min under vacuum (0.13 mbar) at 120 °C (oil-bath temperature), giving 2.13 g (quantitative yield) of trihexyl tetradecylphosphonium triflate;^1^H-NMR (CD_3_CN), δ, ppm: 0.84–0.94 m (12H, 4CH_3_), 1.24–1.37 m (32H, 16CH_2_), 1.37–1.57 m (16H, 8CH_2_), 1.99–2.09 m (8H, 4CH_2_); ^19^F-NMR (CD_3_CN), δ, ppm: −78.0 s (3F, SCF_3_); ^31^P{^1^H}-NMR (CD_3_CN), δ, ppm: 33.5 s.

Method (**b**): A mixture of trihexyl tetradecylphosphonium chloride (1.64 g, 3.16 mmol) and trimethylsilyl triflate [CF_3_SO_2_OSi(CH_3_)_3_, 0.91 g, 4.09 mmol) was stirred at room temperature for 4 h. The residue was dried for 30 min under vacuum (0.13 mbar) at 120 °C (oil-bath temperature) to give 2.00 g (a quantitative yield) of the title compound, which was characterised by NMR spectroscopy. The spectra were identical to those of the product obtained by Method (**a**).

#### *N"-Ethyl-N,N,N',N'-tetramethylguanidinium Trifluoromethanesulfonate* [(CH_3_)_2_NC(NHC_2_H_5_)N(CH_3_)_2_] [CF_3_SO_3_]

Freshly distilled ethyl trifluoromethanesulfonate (870 g, 4.88 mol) was dropped slowly into magnetically stirred *N*,*N*,*N'*,*N'*-tetramethylguanidine (560 g, 4.86 mol) during 9 h at 0 °C. Stirring (1,000 rpm) was continued for 12 h at 30 °C to complete the reaction. Finally the excess of ethyl trifluoromethanesulfonate (4 g, 0.02 mol) was evaporated at 0.01 mbar and 25 °C. The product (1,426 g, 4.86 mol, quantitative yield) was obtained as a clear and colourless liquid; [(CH_3_)_2_NC(C_2_H_5_NH) N(CH_3_)_2_][CF_3_SO_3_]: ^1^H-NMR (CD_3_CN), δ, ppm: 1.12 t (3H, ^3^*J*_H,H_* = * 7.1 Hz, CH_2_CH_3_), 2.88 s (12H, 2[N(CH_3_)_2_]), 3.25 q (2H, , ^3^*J*_H,H_* = * 7.1 Hz, CH_2_CH_3_), 6.46 s (1H, NH); ^19^F-NMR (CD_3_CN), δ, ppm: −79.1 s (3F, CF_3_S); ^13^C-NMR (CD_3_CN), δ, ppm: 12.1 q (CH_2_CH_3_), 39.1 q (N(CH_3_)_2_), 43.2 t (CH_2_CH_3_), 120.8 q (CF_3_S), 163.0 s; [(CH_3_)(CH_2_CH_3_)NC(CH_3_NH)N(CH_3_)_2_][CF_3_SO_3_]: ^1^H-NMR (CD_3_CN), δ, ppm: 1.20 t (3H, ^3^*J*_H,H_* = * 7.1 Hz, CH_2_CH_3_), 2.92 s (12H, [N(CH_3_)_2_]), 3.25 q (2H, ^3^*J*_H,H_* = * 7.1 Hz, CH_2_CH_3_), 6.46 s (1H, NH); ^19^F-NMR (CD_3_CN), δ, ppm: −79.1 s (3F, CF_3_S); ^13^C-NMR (CD_3_CN), δ, ppm: 14.2 q (CH_2_CH_3_), 38.9 q (N(CH_3_)_2_), 39.7 t (CH_2_CH_3_), 120.8 q (CF_3_S), 161.3 s; The ratio of isomers [(CH_3_)_2_NC(C_2_H_5_NH)N(CH_3_)_2_]: [(CH_3_)(CH_2_CH_3_)NC(CH_3_NH)N(CH_3_)_2_] was 2.3:1; Anal. Calcd. for C_8_H_18_O_3_N_3_F_3_S: C, 32.76; H, 6.19; N, 14.33; S, 10.93. Found: C, 32.72; H, 6.67; N, 15.35; S, 10.90.

#### *N,N,N',N'-Tetramethyl-O-methylisouronium Trifluoromethanesulfonate* [(CH_3_)_2_NC(OCH_3_)N(CH_3_)_2_] [CF_3_SO_3_]

Freshly distilled methyl trifluoromethanesulfonate (765 g, 4.66 mol) was dropped slowly into magnetically stirred *N*,*N*,*N'*,*N'-*tetramethylurea (534 g, 4.60 mol) over a period of 9 h at 20 °C. Stirring (1,000 rpm) was continued for 12 h at 25 °C to complete the reaction. Finally the excess of methyltrifluoromethanesulfonate (10 g, 0.06 mol) was evaporated at 0.01 mbar and 25 °C. The product (1,288 g, 4.60 mol, quantitative yield) was obtained as a clear and colourless liquid; [(CH_3_)_2_N)_2_ COCH_3_][CF_3_SO_3_]: ^1^H-NMR (CD_3_CN), δ, ppm: 3.11 s (12H, 2[N(CH_3_)_2_]), 4.10 s (3H, OCH_3_); ^19^F-NMR (CD_3_CN), δ, ppm: −79.2 s (3F, CF_3_S); ^13^C-NMR (CD_3_CN), δ, ppm: 40.7 q (N(CH_3_)_2_), 62.9 q (OCH_3_), 120.8 q (CF_3_S); Anal. Calcd. for C_7_H_15_O_4_N_2_F_3_S: C, 30.00; H, 5.39; N, 10.00; S, 11.44. Found: C, 29.64; H, 5.34; N, 9.92; S, 10.93.

## 4. Conclusions

New economical syntheses of methyl and ethyl triflate from dimethyl- and diethyl carbonate and triflic acid has been developed. These chemicals were used in solvent and halogen free syntheses of high purity triflate ionic liquids via direct alkylation of organic bases: amines, phosphines or heterocyclic compounds. Ionic liquids with the triflate anion possess high thermal and electrochemical stability and fairly low viscosity.
